# The New Dentistry

**Published:** 1914-07-15

**Authors:** Edward H. Baker

**Affiliations:** Chicago, Ills.


### THE NEW DENTISTRY.

BY EDWARD H. BAKER, B. A., M. D., CHICAGO, ILLS.

In the evolution of human understanding, we find that the first manifestation of a dawing intelligence is to do something, to make something. A little child is delighted with its first efforts. The savage feels great pride in his crude implements, pottery and decorations. But his most finished efforts compare with those of the skilled artisan of civilization about as sloyd compares with manual training. One is the cut and try method and its results are inaccurate and irregular; while the other is a definite procedure done by rule and characterized by regular form and close-fitting joints.

But with the growth of civilization and increase of knowledge, the idea of *not* doing certain things attains increasing importance. This is peculiarly true since knowledge has begun to assume scientific form and adopt scientific methods of measurement and investigation.

Dentistry offers no exception to this rule. Within the past ten years, research in bacteriology and pathology have cleared up much of the sequence and mode of action of disease in the human body.

While as yet we are ignorant of many facts, evidence is fast accumulating which points to the mouth as, perhaps, the most important focal-center of infection and disease within the human body. With the growth of this idea, the profession of dentistry suddenly appears in a new role. Instead of being of subsidiary importance, it appears to be of primary importance, and instead of being a calling wherein only mechanical skill and ingenuity are required, it appears to be a profession requiring deep scientific knowledge, not only of the human body and its processes, but of organic and physical chemistry and physics.

There are scarcely any dentists or dental students who realized this fact when they began the study of dentistry. And up to the present time, there has been so little appreciation of the tremendous responsibility for human life and welfare which rests upon the shoulders of the dentist, that dental schools have not yet been able to extend their curriculums to include such medical teaching as will fully enlighten the student as to the injurious and far reaching effects of poor dental work.[1]

Natural contour and contacts, smooth joints, close-fitting bands and even clean teeth—how often are they neglected? Yet we learn that pyorrhea and abscesses, whether open or blind, cause rheumatism, valvular heart-disease, Bright's disease, arterio-sclerosis, liver disease, gallstones, appendicitis, nervous diseases, digestive disorders and nearly all of the chronic ailments that flesh is heir to.[2]

The dentist who makes a poorly fitting crown is, in reality, placing a crown of death upon his patient. Thus the advertising quack, with his $3.00 or $5.00 gold crown, is really a stealthy assassin who, under the guise of saving money for his patient, administers to him a death potion which is as sure as it is slow and unsuspected.

All this is the result of ignorance. The spread of knowledge and of education will do away with most of our ills. While education or training cannot make a good mechanic of one who is entirely lacking in mechanical ability, it can

---

[1]See "A Look Ahead," Editorial by Dr. Edward C. Kirk in the *Dental Cosmos* for April, 1914, 56, 4, p. 497, showing the urgent need of such additions to the curriculum of the Dental School as will span the field between dentistry and medicine.

[2]"The Natural Field of Dentistry," by Edward H. Baker, M. D., *Dental Cosmos*, June, 1912

In this article is a list of over 100 diseases which have been shown to originate in the mouth, with a bibliography of same.

enlighten him and others as to the terrible results of poor work. This knowledge will show him what to refrain from doing, no matter how clumsy he may be with his hands.

Thus even an awkward, poor workman, with little or no mechanical skill, may be led by knowledge to either mercifully abandon his mistaken choice of a profession for one in which he will be less likely to do harm, or else to limit his efforts to operations where he will not injure the health and life of the patient.

Thus we see that the effect of scientific knowledge is to teach us what not to do, even more than what to do. Interference with nature's life processes is dangerous. It is slowly percolating into our minds that from a standpoint of life and health, while fine dental work is of the greatest value to man, poor dental work is a great menace. Instead of the dentist being merely a mechanic, he is a man who is responsible for the lives and health of the inhabitants of the civilized world.

There is another consideration which we must not forget. While teaching as to the far-reaching, injurious effects of bad dentistry is being neglected, so is the teaching of the wonderful curative effects of good dentistry being neglected.

Dentistry is affected in two ways by these omissions. First, the dentist does not realize what great good he is doing his patients and, secondly, as a direct result, the patient must remain in ignorance of the real value of the dentist's services.

To explain my meaning, let us take a concrete example.

A patient comes to a dentist. His mouth is in very bad condition. He is unable to chew, has pyorrhea and a very foul mouth. He is pale and emaciated, has digestive disturbances, perhaps stiffness and rheumatism and many other symptoms.

The dentist puts his mouth in first-class condition. The patient gains 20 or 30 pounds, feels like a new man and pays the dentist his fee. This dentist may or may not get other patients through this patient or his family.

Here is where dentists make a fatal blunder. They do not diagnose (or have someone else to do it for them) the exact physical condition of their patients before and after the dental work is done.

Is the patient anemic? Make a blood count and find out. A blood examination made after the dental work is finished will prove the wonderful change which has occurred. Has the patient chronic Bright's disease? Take his blood-pressure and examine the saliva and urine and find out. Then, after the dentist has completed his work, let him show by successive examinations, made every month, the steady improvement in the patient's condition. He may get entirely well.

Has the patient diabetes? Make a urinalysis test and find out. Then make subsequent tests after the pyorrhea is under control and show the diminution of sugar. It may disappear entirely and permanently.

Has the patient a very high blood pressure? If subsequent tests taken after the dental work is completed show a marked reduction in blood-pressure, it means that the bad mouth condition was causing arterio-sclerosis (hardening of the arteries) or Bright's disease, and that the dental work is going to lengthen that patient's life by many years. These patients will talk freely and convincingly to all their friends.

Will the physician object? Not he. As soon as he finds that the dentist knows how to make diagnostic tests even more accurately than he does and is making them on all his patients, he will pay some attention to what the dentist says, because the dentist now speaks in terms of facts, not vague generalities.

Instead of saying the patient was "run down" or "in bad shape," or "complaining of ill-health," the dentist says:

"The systolic blood-pressure was 159. It is now 128.

"The diastolic pressure was 93. It is now 90.

"The pulse pressure was 66. It is now 38.

"The amount of hemoglobin was 65%. It is now 94%.

"The number of red cells was 3,650,000. It is now 4,830,000.

"The 24-hour specimen of urine showed a little albumin and a number of hyaline and a few granular casts at each examination. It now shows no albumin, no granular casts and only an occasional hyaline cast."

Thus the physician will at once acquire confidence in the dentist because of his evident accuracy and knowledge of medical diagnosis. The physician will be astonished and pleased when he learns what a wonderful effect mouth conditions have on bodily health. He will at once recall cases which he wishes to send to this dentist for attention. He will begin to examine the mouths of his patients and to note pyorrhea. Soon he will be sending *all* his patients to the dentist to have thorough dental attention including prophylaxis, instead of sending an occasional one to have a tooth pulled. To which dentist will he send his patients? To those dentists whose scientific knowledge he respects—to those who are familiar with medical diagnosis.

Thus, this diagnosis will result in securing the co-operation and respect of the physician. It will increase the demand for dental services many times in a given locality. It will necessarily increase dental fees. It will make dentistry the "Greatest Profession in the World."

The dentist is often in a better position to make a diagnosis than the physician is, for people who consider themselves well and in no need of the services of a physician, go to a dentist. And the dentist will often be led by the appearance of the gums or saliva, to suspect a physical condition unknown to the patient or his physician. In these cases as well as in those of his other patients, the taking of blood pressure and examination of saliva, urine and blood will reveal the facts regarding grave physical disorders.

The dentist who discovers incipient Bright's disease, Diabetes, etc., in an incipient stage, when curative measures will, perhaps, arrest its progress, is certainly a friend to humanity and will be recognized as such both by patients and physicians.

Thus the dentist has a natural field of diagnosis which is not occupied by the physician and he should not neglect his opportunities and responsibilities in this field.

The teaching of physical and clinical diagnosis appears to be most needed in the practice of dentistry today. For this reason, this Institute has devoted a large amount of time and effort to these subjects in the curriculum of its Dental Study Classes.

It seems to the writer that even when the dental college course is extended to four years, there will still be need of post-graduate work, because advances in science, dentistry and medicine are being made with such rapidity that a man who has been out in practice five or ten years has much to learn.

Since we have discovered that the field of dentistry is the most important one as regards the health and life of the people, it is no longer permissible for a dentist to consult his own preferences as to whether or not he wishes to keep abreast of the scientific knowledge of the day. Human life and human welfare demand that he must either do his best work, or resign and seek some other field where mediocre efforts are not so dangerous to mankind.

For the first time in the history of the world, human life is beginning to be considered of some importance. The "safety first" movement is here and is spreading. Factories, tenements, theatres, steamships and railroads are adopting pracautionary safety measures, because it is slowly filtering into our minds that the terrible waste of human life is needless. It is going to be the same with sickness. Our growing knowledge as to the causation, progress and sequence of disease shows that mouth conditions probably surpass every other factor in importance.

Hence, in the evolution of human society, the profession of dentistry is about to become the most important, the best rewarded and the greatest profession in the world.

Every dentist should be proud of the privilege of being a member of the dental profession today and of taking part in its new and glorious future career which is now opening up to view. But not all will avail themselves of this privilege. Some will cling to past traditions with their lack of ideals and responsibilities.

The "dentistry of to-morrow" carries with it great responsibilities, for not only will the dentist know of the great importance of his own operations, but he will be the great health teacher. Thus, in reality, he will be the keeper of public health.

Dr. C. N. Johnson has recently called attention[3] to the danger that physicians might overestimate the evils arising from bad or doubtful dentistry. (For in this transition period in medicine and dentistry, we do not yet know, or see clearly what is good and what is bad—which methods are beneficial and which are injurious.)

There is no doubt a general tendency of the pendulum to swing to extremes at first, and we cannot expect physicians or laymen to offer any exception to this rule in the present instance. Dentistry will doubtless receive some undeserved criticism. On the other hand, there is only one practical method of avoiding this criticism, and that is, to adopt the method of diagnosing cases before and after dental operations.

When physicians and the public realize how much good the dentists are doing, they will praise them instead of blaming them. At least, they will praise those practitioners who are accustomed to make these diagnoses, and who thus give practical evidence of their appreciation of the importance of mouth conditions as related to the health of the rest of the body.

I consider that at present the only safety to dentistry lies in advancing and in gladly accepting the new responsibilities which are so suddenly and unexpectedly thrust upon it. The reactionists, the more conservative practitioners, who are slow to reach a conclusion, are the ones who are most likely to call down criticism upon the entire profession.

---

[3] *Dental Review*, 28, 4, April, 1914, p. 364.

Conservatism is the great safe-guard when it is backed up by facts; but when it is founded upon ignorance of facts it is the most dangerous, revolutionary and explosive force in the world.

There are now various diagnostic methods which are sufficiently simple and easy of acquiring a technique, so that in a short time a dentist can learn to diagnose some of the most dangerous conditions of bodily disease with comparative ease. Even if his office-girl did most of the work, he should be able to make an intelligent interpretation of a blood-count sheet, an urinalysis, or a blood-pressure determination. With a little experience and practice, he would soon attain a degree of knowledge and skill which would make the daily practice of dentistry an ever-changing field of joyful interest instead of a dull, uninteresting, humdrum round of toil, the monotony of which gets upon his nerves and takes years away from his life.—*Dental Review*, June, 1914.

